# The Reduction of Regional Cerebral Blood Flow in Normal-Appearing White Matter Is Associated with the Severity of White Matter Lesions in Elderly: A Xeon-CT Study

**DOI:** 10.1371/journal.pone.0112832

**Published:** 2014-11-17

**Authors:** Jianhui Fu, Jie Tang, Jinghao Han, Zhen Hong

**Affiliations:** 1 Department of Neurology, Huashan Hospital, Fudan University, Shanghai, China; 2 Department of Neurology, Shanghai Pudong Hospital, Fudan University Pudong Medical Center, Shanghai, China; 3 Departments of Medicine and Therapeutics, Chinese University of Hong Kong, Prince of Wales Hospital, Hong Kong, China; University of Jaén, Spain

## Abstract

White matter lesions (WMLs) in normal elderly are related to chronic ischemia, and progression of WML occurs mostly in moderate to severe disease. However, the mechanism is uncertain. Thus, we enrolled fifty-six normal elderly patients without large artery disease. The severity of WML on MRI was graded as grade 0, I, II and III using the modified Fazekas scale. Cerebral blood flow (CBF) was measured by Xenon-CT. We found that CBF (mL/100 g/min) within periventricular lesions and in the right and left centrum semiovales were 20.33, 21.27 and 21.03, respectively, in group I; 16.33, 15.55 and 15.91, respectively, in group II; and 14.05, 14.46 and 14.23, respectively, in group III. CBF of normal-appearing white matter (NAWM) around periventricular areas and in the right and left centrum semiovales were 20.79, 22.26 and 22.15, respectively, in group 0; 21.12, 22.17 and 22.25, respectively, in group I; 18.02, 19.45 and 19.62, respectively, in group II; and 16.38, 18.18 and 16.74, respectively, in group III. Significant reductions in CBF were observed not only within lesions but also in NAWM surrounding the lesions. In addition, CBF was reduced significantly within lesions compared to NAWM of the same grade. Furthermore, CBF was reduced significantly in NAWM in grades II and III when compared to grades 0 and I. Our finding indicates that ischemia may play a role in the pathogenesis of WML. Additionally, our finding provides an alternative explanation for finding that the progression of WML occurred more commonly in patients with moderate to severe WML.

## Introduction

White matter lesions (WMLs) are frequently observed on Flair and T2-weighted brain MRI scans in clinically normal elderly patients and are strongly associated with increasing age [1], [2]. Clinical evidence has shown that WMLs are related to a variety of neurological diseases, especially vascular cognitive impairment [3]–[5]. Anatomical and pathological studies have suggested that chronic ischemia caused by diffuse arteriolosclerosis may contribute to the presence of WML [6]–[9]. Many studies have found significant reduction in blood flow within the WMLs by measuring cerebral blood flow (CBF) using different technique [10]–[14]. However, whether a reduction of blood flow is the cause of WML or just a secondary response to the reduced demands of damaged tissue is still unknown.

Using Flair and T2-weighted brain MRI, O’Sullivan et al [11] were the first to report a reduction in CBF in both periventricular WMLs and normal–appearing white matter (NAWM) around periventricular areas. This finding further suggests that chronic ischemia may be the cause of WMLs. If this is the case, areas with NAWM with decreased CBF may develop into WML in long-term follow-up studies. However, there are some limitations in this study that may weaken these important findings. First, all participants had a history of clinical lacunar syndrome, which may be caused by a sudden disruption of blood supply in one of the perforating arteries. Diagnosis of intracranial large artery disease was based on the diameter of the acute infarct on MRI; hence, intracranial large artery disease cannot be completely ruled out. Second, the MMSE score of some participants was less than 24; thus, patients with dementia, including Alzheimer disease, may have been enrolled. Third, the relatively small sample size limited classification of participants by the severity of WML, and as a result, the relationship between the CBF in NAWM regions and WMLs and the severity of the WML is not clear.

More recent longitudinal studies [15]–[17] have shown that the progression of WML occurred mostly in moderate to severe WML during the following period. All those findings indicated that the severity of WML was associated with the progression of WML. However, the mechanism is uncertain.

Chronic ischemia is the underlying cause of WML; therefore, our hypothesis is that blood flow is decreased not only in white matter lesions but also in areas of NAWM around the lesions and that the reduction of the blood flow in both areas is associated with the severity of WML. To test this hypothesis, we measured CBF within white matter lesions and in normal-appearing white matter around the lesions by Xeon-CT in normal elderly subjects, which may provide further evidence regarding the role of chronic ischemia in the pathogenesis of WML.

## Methods

### Participants and Data collection

One hundred eighty-three consecutive subjects who visited the outpatient clinic of the neurological department for general health check at Huashan Hospital, Shanghai, China, between October 2006 and June 2007 were studied. All subjects underwent a careful neurological and general physical examination. A history of hypertension, diabetes mellitus, stroke or transient ischemic attack, ischemic heart disease, atrial fibrillation, smoking, and alcohol consumption were also noted. Detailed information on risk factors has been reported previously. This study and its methodology were approved by the Institutional Review Board of Huashan Hospital and by the ethics committee of Huashan Hospital.

### Inclusion and Exclusion Criteria

Subjects were enrolled in this study if the following criteria were fulfilled: (1) no history of stroke and atrial fibrillation, (2) MMSE*>*24, and (3) no signs or symptoms of neurological manifestations when examined by a neurologist. Subjects younger than 60 years of age or with a history of head trauma or neurosurgery were excluded. Carotid artery ultrasonography was performed in each individual using a color-flow B-mode Doppler ultra-sonography (ATL3000, USA) with a 7.5-MHz imaging transducer. Carotid artery stenosis was defined according to standardized criteria. Subjects with carotid stenosis of *>*50% lumen diameter reduction were excluded. MR angiography was performed for each individual to rule out intracranial artery stenosis that might interfere with the hemodynamic status. A total of 56 subjects were included in the study and underwent further neuroimaging examinations. The mean age of all participants was 67.4±8.2 (range, 60–79) years, and 34 (60.7%) were men. Each subject signed an informed consent form.

### MRI Examinations

Brain MRI examination was performed with a 1.5 T scanner (GE Signa Horizon) using a head coil with quadrature detection. The brain imaging protocol involved (1) T1- and T2-weighted spin-echo axial images (TR/TE 440/14 ms and TR/TE/3000/110 ms, respectively) and sagittal T1-weighted images (2) fluid attenuated inversion recovery (FLAIR) axial images (TR/TE 10002/126 ms). All axial images had a 5-mm slice thickness with a 0.5-mm slice gap and a matrix of 256×205.

WMLs were detected as hyperintensity on FLAIR sequence of MRI, without prominent hypointensity on T1-weighted scans, and they were 2 mm or more in diameter on hard copy film. The lesions were classified into four grades according to the modified Fazekas rating scale [18]: grade 0, no lesion (including symmetrical, well defined caps or bands); grade I, focal lesions >2 mm; grade II, beginning confluence of lesions; and grade III, diffuse involvement of the entire region, with or without involvement of U fibers.

### Xenon Contrast CT Examinations

Xenon CT was performed within 30 days of the MRI examinations. To match the slices of CT and MR imaging, the CT table was adjusted to the same slice width as that of the MR imaging. An initial conventional CT scan was executed. We examined 4 CT sections that showed that WML matched with MRI on xenon contrast CT scan. Each participant inhaled a gas mixture containing 28% stable xenon, 25% oxygen, and 47% air for 4.3 minutes. For each section, CT scans were performed twice before the xenon inhalation and 4 times during the xenon inhalation. At the end of the study, the results were transferred to a workstation. Data analysis was performed with dedicated post-processing software (Xe-CT System 2; Diversified Diagnostic Products Inc, Houston, TX).

### Analysis of Regional CBF

To analyze regional CBF, the whole brain was divided into cortex, white matter and basal ganglia. The cortex was then subdivided into frontal, parietal, occipital and temporal lobes. The white matter region was subdivided into centrum semiovale and periventricular regions (including anterior, lateral and posterior regions of ventricle). The basal ganglia were subdivided into caudate nucleus, lenticular nucleus and thalamus.

**Figure 1:**
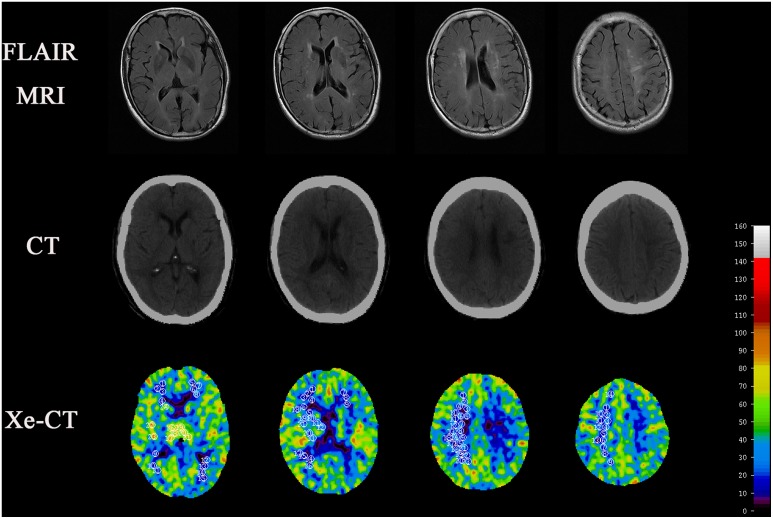
Flair imaging, normal CT and Xe-CT at different levels of subjects with WML.

To calculate the values of CBF in each region of white matter and basal ganglia, a round ROI (region of interest) of 40 pixels was selected as a measurable unit. We placed the round ROI on each distinct WML or normal-appearing white matter region separately at each section level in each individual, and the mean value of CBF was then calculate for each region (Figure 1). The mean values of CBF in each region of the cortex were calculated automatically by the software. CBF measurement was carried out by the same radiologist.

### Statistical Analysis

Statistical analysis software (SAS) 6.12 was used for the statistical analysis. Statistical significance for intergroup differences was assessed by the 2-tailed Fisher’s exact test, chi-squared test for categorical variables and Student’s t-test for continuous variables. A level of P<0.05 was considered statistically significant.

## Results

Of the 56 cases, 10 had grade 0 WML, 13 had grade I lesions, 12 had grade II lesions and 16 had grade III lesions. The clinical characteristics of all participants with and without WML are shown in table 1. We did not find any significant differences in age, gender or other vascular risk factors among the different grades of WML.

**Table 1:** Clinical characteristics of all participants (n = 56) in each grade.

Clinical characteristics	Grade 0 (n = 10)	Grade I (n = 13)	Grade II (n = 17)	Grade III (n = 16)	P*
Age, years, Mean (SD)	65.3±6.3	64.5±5.8	68.9±7.7	68.1±8.1	0.310
Male, n	7	8	11	8	0.672
Hypertension, n	4	5	9	10	0.434
Diabetes mellitus, n	1	1	1	4	0.211
Ischemic heart disease, n	1	0	2	0	0.168
Smoking, n	0	1	1	3	0.269
Alcohol consumption, n	0	0	1	1	0.662

*Compared among the four groups.

The mean values of CBF within periventricular lesions and in the right and left centrum semiovales among different WML grades are shown in table 2. The mean CBF (mL/100 g/min) within lesions in these areas were 20.33 (2.52), 21.27 (1.02) and 21.03 (1.83), respectively, in group I; 16.33 (2.03), 15.55 (1.71) and 15.91 (0.98), respectively, in group II; and 14.05 (2.63), 14.46 (2.17) and 14.23 (1.95), respectively, in group III. Group differences in terms of CBF between different severities of WML were significant (P<0.05) within most WML regions.

**Table 2:** Mean (SD) CBF (mL/100 g/min) of NAWM and lesions in each grade.

Region	Grade 0 (n = 10)	Grade I (n = 13)			Grade II (n = 17)			Grade III (n-16)		
	NAWM	NAWM	Lesions	P*	NAWM	Lesions	P*	NAWM	Lesions	P*
RLV	21.58±2.33	21.53±2.14	20.67±1.84	0.006	18.52±1.17^1^	17.18±2.42^3^	<0.001	16.80±2.48^1^^,^ 2	14.90±2.76^3^^,^ 4	<0.001
LLV	23.10±4.31	23.01±4.15	22.14±3.82	0.002	18.82±2.56^1^	16.72±1.84^3^	<0.001	17.21±5.93^1^	13.61±3.51^3^^,^ 4	<0.001
RAV	19.97±3.72	20.84±3.44	20.28±2.62	0.247	18.03±3.12^1^	16.52±2.24^3^	0.005	16.13±3.12^1^	13.97±2.19^3^^,^ 4	0.004
LAV	19.81±3.48	21.67±3.59	20.80±2.72	0.053	17.17±2.47^1^	15.19±1.91^3^	<0.001	16.08±2.96^1^	13.66±2.64^3^	<0.001
RPV	19.39±2.26	19.72±2.29	18.48±2.13	0.224	18.84±2.57	17.53±2.19	<0.001	16.83±2.45^1^^,^ 2	14.25±2.29^3^^,^ 4	<0.001
LPV	20.88±2.54	19.97±2.06	19.61±1.98	0.172	16.72±2.56^1^	14.86±1.55^3^	0.001	15.23±2.38^1^	13.90±2.40^3^	<0.001
WPV	20.79±2.78	21.12±2.95	20.33±2.52	0.106	18.02±2.41^1^	16.33±2.03^3^	<0.001	16.38±3.22^1^^,^ 2	14.05±2.63^3^^,^ 4	<0.001
RCS	22.26±1.9	22.17±1.50	21.27±1.02	0.017	19.45±1.94^1^	15.55±1.71^3^	<0.001	18.18±2.84^1^	14.46±2.17^3^	<0.001
LCS	22.15±2.4	22.25±2.13	21.03±1.83	0.003	19.62±1.54^1^	15.91±0.98^3^	<0.001	16.74±2.97^1^^,^ 2	14.23±1.95^3^^,^ 4	<0.001

P* refers to CBF within lesions compared that in NAWM in the same group.

1 Compared with grades 0 and I, CBF of NAWM in grades II and III, P value <0.05;

2 compared with grade II, CBF of NAWM in grade III, P value <0.05.

3 Compared with grade I, CBF within lesions in groups II and III, P value <0.05;

4 compared with grade II, CBF within lesions in group III, P value <0.05.

NAWM means normal-appearing white matter. RLV means right lateral of ventricle, LLV means left lateral of ventricle; RAV means right anterior of ventricle, LAV means left anterior of ventricle; RPV means right posterior of ventricle, LPV means left posterior of ventricle; WPV means whole periventricular areas; RCS means right centrum semiovale, LCS means left centrum semiovale.

The mean values of CBF in the periventricular NAWM regions and in right and left centrum semiovales among different grades of WML are shown in table 2. The mean CBF (mL/100 g/min) of NAWM in these areas were 20.79 (2.78), 22.26 (1.9) and 22.15 (2.4), respectively, in group 0; 21.12 (1.50), 22.17 (1.50) and 22.25 (2.13), respectively, in group I; 18.02 (2.41), 19.45 (1.94) and 19.62 (1.54), respectively, in group II; and 16.38 (3.22), 18.18 (2.84) and 16.74 (2.97), respectively, in group III. There is no significant difference in CBF values of NAWM between grade 0 and I. but significant differences (P<0.05) in CBF were found in subjects with grade II and III WMLs when compared to those with grade 0 and I WMLs. And the differences in CBF of NAWM between grades II and III WMLs were also significant (P<0.05).

A comparison of CBF between NAWM and lesions in the same grade WML is shown in table 2. A significant reduction in CBF was noted in all regions with grade II and III WML when compared with the corresponding areas of NAWM (P<0.05). A significant reduction in CBF was also found in some regions with grade I WML when compared with that of NAWM.

A comparison of CBF between different regions in the same grade WML or in the NAWM around the same grade WML is shown in table 3. There were not significant differences (P>0.05) in CBF between right or left centrum semiovale area and periventricular area in each grade WML or in the NAWM around the grade I or III WML.

**Table 3:** Mean (SD) CBF values (mL/100 g/min) in different area in each grade.

	Region	RCS	WPV	P*	LCS	WPV	P*
Grade I (n = 13)	NAWM	22.17±1.50	21.12±2.95	0.095	22.25±2.13	21.12±2.95	0.026
	Lesions	21.27±1.02	20.33±2.52	0.075	21.03±1.83	20.33±2.52	0.107
Grade II (n = 17)	NAWM	19.45±1.94	18.02±2.41	0.004	19.62±1.54	18.02±2.41	0.007
	Lesions	15.55±1.71	16.33±2.03	0.067	15.91±0.98	16.33±2.03	0.242
Grade III (n = 16)	NAWM	18.18±2.84	16.38±3.22	0.010	16.74±2.97	16.38±3.22	0.604
	Lesions	14.46±2.17	14.05±2.63	0.277	14.23±1.95	14.05±2.63	0.729

P* refers to CBF comparison between different areas in the same group. NAWM means normal-appearing white matter, WPV means whole periventricular areas; RCS means right centrum semiovale, LCS means left centrum semiovale.

The mean CBF values in various grey matter regions are shown in table 4. Significant reductions in CBF of the bilateral temporal lobes and the lenticular nucleus were observed in grades II and III when compared with CBF of grades 0 and I (P<0.05). CBF in other grey matter regions and nuclei did not show significant differences when compared to the flow within WMLs of different severities.

**Table 4:** Mean (SD) CBF values (mL/100 g/min) of grey matter regions in each grade.

Gray matter region	Grade 0 (n = 10)	Grade I (n = 13)	Grade II (n = 17)	Grade III (n = 16)	P
R frontal lobe	48.00±10.62	51.67±10.83	46.58±14.48	46.48±10.71	0.448
L frontal lobe	47.46±10.65	50.39±10.33	43.39±15.56	42.34±10.13	0.192
R temporal lobe	53.04±7.71	56.06±10.33	48.92±15.56^1^	47.21±10.13^1^	0.010
L temporal lobe	50.76±7.02	53.80±5.59	45.86±8.07^1^	43.29±9.86^1^	0.004
R parietal lobe	48.85±11.17	51.83±14.21	45.99±14.82	47.31±9.99	0.469
L parietal lobe	45.49±8.44	48.43±10.10	42.87±12.9	42.60±11.02	0.330
R occipital lobe	46.16±6.73	47.88±7.05	43.98±8.72	44.29±8.06	0.370
L occipital lobe	44.58±6.11	45.53±7.19	44.08±9.14	41.72±7.29	0.435
R caudate nucleus	71.99±12.16	74.66±9.29	68.92±10.75	66.78±17.89	0.283
L caudate nucleus	69.61±7.55	67.91±11.16	65.51±11.77	60.28±10.28	0.214
R lenticular nucleus	72.67±14.82	79.37±10.57	67.96±13.45^1^	67.54±11.15^1^	0.027
L lenticular nucleus	78.61±10.07	81.29±6.99	75.70±12.38^1^	72.17±11.62^1^^,^ 2	0.032
R thalamus	83.34±12.25	84.26±12.56	79.80±10.72	80.01±11.91	0.533
L thalamus	83.29±12.41	84.17±11.55	81.94±9.16	81.46±12.88	0.339

1 Compared with grades 0 and I, CBF in grades II and III, P value <0.05;

2 compared with grade II, CBF in grade III, P value <0.05. R means right side, L means left side.

## Discussion

Our data showed that the regional cerebral blood flow in white matter lesions and in normal-appearing white matter was decreased in normal elderly with white matter lesions. Moreover, the degree of the decrease was related to the severity of the lesions.

Our study showed ischemia within WMLs, which is consistent with other studies using a variety of techniques to measure CBF [10]–[14], [19]. Furthermore, reductions of CBF within lesions were associated with the severity of WMLs in both the centrum semiovale and the periventricular regions. Using xenon contrast CT methods, Miyazawa et al [10] found a similar reduction in CBF in the centrum semiovale in a group of neurological normal elderly without large artery disease. But they did not measure the CBF within WMLs in the periventricular regions because subjects with periventricular WMLs were excluded in their study. Hatazawa et al [13] found a reduction in blood flow within the WML using the positron emission tomographic (PET). However, there was no significant difference in CBF between subjects with severe and mild WML. The limitations of the study included a relatively small sample size and poor spatial resolution of PET to differentiate normal and abnormal white matter, which may underestimate the reduction of cerebral blood flow.

The most important finding of our study is that the reduction of CBF is not only in WML areas but also in NAWM areas around the lesion, and the CBF in WML is lower than in NAWM areas around it. Using 1.5 T MRI and 99 mTc single-photo emission computed tomography (SPECT), a recent study [19] found that the WMH perfusion was lower than NAWM perfusion in normal elderly, which is consistent of our results. Using Flair and T2-weighted brain MRI, O’Sullivan [11] also found a reduction of CBF in the periventricular lesions when compared to areas with normal-appearing white matter. However, a similar reduction in CBF was not observed in the centrum semiovale, and the reduction in CBF in NAWM regions was not associated with the severity of WML. Recent study found that microstructural deterioration within NAWM in cerebral small vessel diseases by DTI [20], and the decrease of CBF in NAWM exhibited in our results may be a possible explanation of this finding. Therefore, combining the DTI and flair images can better assess the white matter changes [21]. Our finding of reduced CBF in both WML and NAWM regions provides further evidence that chronic ischemia may play a role in the pathogenesis of WML in the centrum semiovale and periventricular regions.

Neuro-epidemiological studies showed that the progression of WMLs occurred mostly in moderate to severe WML but seldom in mild WML during the follow-up period [15]–[17]. In our study, The CBF in NAWM surrounding grades II and III WML areas was substantially lower than that surrounding grades 0 and I WML areas, which means that even look like normal form MRI imaging, in fact, the hypoperfusion in areas around WML in grades II and III is serious. Recently, a prospective study showed that the changes of NAWM precede visually appreciable WML that was only the tip of the iceberg of white matter alteration [22]. The significant decline of CBF in NAWM surrounding moderate or severe WML observed by us suggests the grade II or III WML may be more likely to develop than grade I WML. However, the relationship between the CBF in NAWM and the progression of WML needs to be evaluated in longitudinal studies.

Although there has been some controversy regarding whether WML in centrum semiovale and in periventricular regions represent distinct subcategories of WML or should be considered as a continuum [6], [8], [23]–[25], our data indicated that WMLs in the centrum semiovale and in the periventricular regions may share a common underlying chronic ischemic mechanism. We found that reduction of CBF is only associated with the severity but not the location of the lesions. Our results are consistent with evidence from anatomical and pathological studies [6]–[8], [25]. Blood supply in the centrum semiovale and periventricular regions come from long perforating arterioles with few collateral compensation; thus, these areas are considered watershed areas, which are more vulnerable to the assault by hypoperfusion caused by the fluctuation in blood pressure [7]. Pathological studies [6], [8] have also demonstrated that vascular fibrosis and lipohyalinosis were observed in the centrum semiovale and the periventricular regions. DeCarli et al [25] failed to identify distinct subcategories of lesions located in the centrum semiovale and the periventricular regions using a MRI 3D mapping technique. All the aforementioned studies suggest that categorical distinctions between these two regions may be arbitrary.

Notably, in our study, a higher severity of WMLs was associated with lower regional CBF in bilateral temporal lobes and the lenticular nucleus. Reductions of CBF in the basal ganglia have also been reported by a PET study with H_2_^15^O and ^15^O_2_ that was performed in subjects with or without WML [13], but we are the first to show an association between reduced CBF in the temporal lobe and the severity of WML. The values of CBF in each lobe were calculated automatically by the software to avoid methodological bias; thus, the results are reliable. Two points may help explain the association between the severity of WMLs and the decrease of CBF in temporal lobe and lenticular nucleus. First, WML is strongly associated with increasing age and arteriolosclerosis is a systemic disease, we believe that apart from white matter lesions, reduced blood supply in the vascular bed due to brain atrophy may also contribute to the relatively low blood flow. Second, the connectional diaschisis may also an alternative explanation of those results. The cortical-subcortical circuits go though the centrum semiovale and periventricular white matter, and the ischemia in whiter matter may lead to the dysfunction of brain network, which may contribute to connectional diashcisis [26], [27] that further cause the reduction of CBF in remote areas by the impaired neurovascular coupling [28].

The spatial limitation of xenon-CT must be considered when interpreting our results. As the sensitivity of CT in detecting WML is lower than MRI, it is difficult to discriminate normal-appearing white matter from the white matter lesions on xenon-CT mapping completely. The drawing of ROI may be arbitrary to some extent even though we compared the lesions on MRI and CT carefully before calculating the values of CBF. To minimize the measurement error, all the ROI analyses were completed by the same radiologist.

In summary, we found that the reductions of CBF not only in WML but also in NAWM regions were associated with the severity of WML in the centrum semiovale and the periventricular regions in neurologically normal elderly subjects without large artery occlusive disease. The reduction of CBF in bilateral temporal lobes and the lenticular nucleus further support our hypothesis that chronic ischemia may play a major role in the pathogenesis of WML. Moreover, our findings might provide an alternative explanation for the phenomenon that progression of WML occurs more commonly in patients with moderate to severe WML. Long-term follow-up studies may help to know if NAWM regions with reduced CBF further develop into white matter lesions, and help to generate a causal relationship between chronic ischemia and WML.

## References

[1] Grueter BE, Schulz UG (2012) Age-related cerebral white matter disease (leukoaraiosis): a review. Postgrad Med J 88: 79–87. 22184252 10.1136/postgradmedj-2011-130307

[2] O’Sullivan M (2008) Leukoaraiosis. Pract Neurol 8: 26–38. 18230707 10.1136/jnnp.2007.139428

[3] Nichtweiss M, Weidauer S, Treusch N, Hattingen E (2012) White matter lesions and vascular cognitive impairment: part 1: typical and unusual causes. Clin Neuroradiol 22: 193–210. 22327799 10.1007/s00062-012-0134-5

[4] Poggesi A, Gouw A, van der Flier W, Pracucci G, Chabriat H, et al (2013) Cerebral white matter changes are associated with abnormalities on neurological examination in non-disabled elderly: the LADIS study. J Neurol 260: 1014–1021. 23180181 10.1007/s00415-012-6748-3

[5] Schmidt R, Ropele S, Ferro J, Madureira S, Verdelho A, et al (2010) Diffusion-weighted imaging and cognition in the leukoariosis and disability in the elderly study. Stroke 41: e402–408. 20203319 10.1161/STROKEAHA.109.576629

[6] Fazekas F, Kleinert R, Offenbacher H, Schmidt R, Kleinert G, et al (1993) Pathologic correlates of incidental MRI white matter signal hyperintensities. Neurology 43: 1683–1689. 8414012 10.1212/wnl.43.9.1683

[7] Pantoni L, Garcia JH (1997) Pathogenesis of leukoaraiosis: a review. Stroke 28: 652–659. 9056627 10.1161/01.str.28.3.652

[8] Auriel E, Bornstein NM, Berenyi E, Varkonyi I, Gabor M, et al (2011) Clinical, radiological and pathological correlates of leukoaraiosis. Acta Neurol Scand 123: 41–47. 20219022 10.1111/j.1600-0404.2010.01341.x

[9] Brown WR, Thore CR (2011) Review: cerebral microvascular pathology in ageing and neurodegeneration. Neuropathol Appl Neurobiol 37: 56–74. 20946471 10.1111/j.1365-2990.2010.01139.x PMC3020267

[10] Miyazawa N, Satoh T, Hashizume K, Fukamachi A (1997) Xenon contrast CT-CBF measurements in high-intensity foci on T2-weighted MR images in centrum semiovale of asymptomatic individuals. Stroke 28: 984–987. 9158638 10.1161/01.str.28.5.984

[11] O’Sullivan M, Lythgoe DJ, Pereira AC, Summers PE, Jarosz JM, et al (2002) Patterns of cerebral blood flow reduction in patients with ischemic leukoaraiosis. Neurology 59: 321–326. 12177363 10.1212/wnl.59.3.321

[12] Fu JH, Yuan J, Li S, Guo QH, Hong Z, et al (2009) [Quantitative analysis of regional cerebral blood flow in elderly with white matter lesions]. Zhonghua Yi Xue Za Zhi 89: 1175–1178. 19595081

[13] Hatazawa J, Shimosegawa E, Satoh T, Toyoshima H, Okudera T (1997) Subcortical hypoperfusion associated with asymptomatic white matter lesions on magnetic resonance imaging. Stroke 28: 1944–1947. 9341700 10.1161/01.str.28.10.1944

[14] Uh J, Yezhuvath U, Cheng Y, Lu H (2010) In vivo vascular hallmarks of diffuse leukoaraiosis. J Magn Reson Imaging 32: 184–190. 20578025 10.1002/jmri.22209 PMC3236451

[15] Schmidt R, Enzinger C, Ropele S, Schmidt H, Fazekas F (2003) Progression of cerebral white matter lesions: 6-year results of the Austrian Stroke Prevention Study. Lancet 361: 2046–2048. 12814718 10.1016/s0140-6736(03)13616-1

[16] Firbank MJ, Teodorczuk A, van der Flier WM, Gouw AA, Wallin A, et al (2012) Relationship between progression of brain white matter changes and late-life depression: 3-year results from the LADIS study. Br J Psychiatry 201: 40–45. 22626634 10.1192/bjp.bp.111.098897

[17] Kreisel SH, Blahak C, Bazner H, Inzitari D, Pantoni L, et al (2013) Deterioration of gait and balance over time: the effects of age-related white matter change–the LADIS study. Cerebrovasc Dis 35: 544–553. 23838682 10.1159/000350725

[18] Wahlund LO, Barkhof F, Fazekas F, Bronge L, Augustin M, et al (2001) A new rating scale for age-related white matter changes applicable to MRI and CT. Stroke 32: 1318–1322. 11387493 10.1161/01.str.32.6.1318

[19] Makedonov I, Black SE, MacIntosh BJ (2013) Cerebral small vessel disease in aging and Alzheimer’s disease: a comparative study using MRI and SPECT. Eur J Neurol 20: 243–250. 22742818 10.1111/j.1468-1331.2012.03785.x

[20] Papma JM, de Groot M, de Koning I, Mattace-Raso FU, van der Lugt A, et al (2014) Cerebral small vessel disease affects white matter microstructure in mild cognitive impairment. Hum Brain Mapp 35: 2836–2851. 24115179 10.1002/hbm.22370 PMC6869489

[21] Zhan W, Zhang Y, Mueller SG, Lorenzen P, Hadjidemetriou S, et al (2009) Characterization of white matter degeneration in elderly subjects by magnetic resonance diffusion and FLAIR imaging correlation. Neuroimage 47 Suppl 2: T58–65. 19233296 10.1016/j.neuroimage.2009.02.004 PMC2720418

[22] de Groot M, Verhaaren BF, de Boer R, Klein S, Hofman A, et al (2013) Changes in normal-appearing white matter precede development of white matter lesions. Stroke 44: 1037–1042. 23429507 10.1161/STROKEAHA.112.680223

[23] Sachdev P, Wen W, Chen X, Brodaty H (2007) Progression of white matter hyperintensities in elderly individuals over 3 years. Neurology 68: 214–222. 17224576 10.1212/01.wnl.0000251302.55202.73

[24] Gouw AA, Seewann A, van der Flier WM, Barkhof F, Rozemuller AM, et al (2011) Heterogeneity of small vessel disease: a systematic review of MRI and histopathology correlations. J Neurol Neurosurg Psychiatry 82: 126–135. 20935330 10.1136/jnnp.2009.204685

[25] DeCarli C, Fletcher E, Ramey V, Harvey D, Jagust WJ (2005) Anatomical mapping of white matter hyperintensities (WMH): exploring the relationships between periventricular WMH, deep WMH, and total WMH burden. Stroke 36: 50–55. 15576652 10.1161/01.STR.0000150668.58689.f2 PMC3816357

[26] Carrera E, Tononi G (2014) Diaschisis: past, present, future. Brain. 10.1093/brain/awu101 24871646

[27] Lawrence AJ, Chung AW, Morris RG, Markus HS, Barrick TR (2014) Structural network efficiency is associated with cognitive impairment in small-vessel disease. Neurology. 10.1212/WNL.0000000000000612 PMC4115608 24951477

[28] Hall CN, Reynell C, Gesslein B, Hamilton NB, Mishra A, et al (2014) Capillary pericytes regulate cerebral blood flow in health and disease. Nature 508: 55–60. 24670647 10.1038/nature13165 PMC3976267

